# Evaluation of the Breastfeeding Dynamics of Neonates with Ankyloglossia via a Novel Ultrasonographic Technique

**DOI:** 10.3390/diagnostics13223435

**Published:** 2023-11-13

**Authors:** Arzu Alan, Ayse Isil Orhan, Kaan Orhan

**Affiliations:** 1 Ankara 75th Year Oral and Dental Health Hospital, Ministry of Health, Ankara 06230, Türkiye; arzudogruyol@yahoo.com; 2 Department of Pediatric Dentistry, Faculty of Dentistry, Ankara Yildirim Beyazit University, Ankara 06220, Türkiye; isilcihan@yahoo.com; 3 Department of Dentomaxillofacial Radiology, Faculty of Dentistry, Ankara University, Ankara 06560, Türkiye

**Keywords:** breastfeeding, ankyloglossia, ultrasonography, diagnostic imaging, tongue movement, sucking function

## Abstract

To effectively address breastfeeding issues for neonates and mothers, one must understand the physiology of breastfeeding and the anatomical components involved in sucking, swallowing, and respiration. This study compared the tongue position and movement of neonates with tongue ties versus healthy controls during sucking. A new objective ultrasonography diagnostic approach was also introduced for the orofacial region. This retrospective study evaluated B-mode and M-mode ultrasonography images from 30 neonates clinically diagnosed with tongue tie, and a control group of 30 neonates. B-mode ultrasound images were used to examine several characteristics to locate the nipple in the oral cavity during breastfeeding. Anatomic M-mode ultrasound images were used to assess tongue movement during sucking. The nipple moved farther from the intersection of the hard and soft palates during the sucking cycle in the ankyloglossia group than in the control group (*p* < 0.05). Compared to the control group, neonates with ankyloglossia have a lower capacity to lift the anterior tongue toward the palate when sucking (*p* < 0.05). There was no significant difference in tongue movement metrics between the two groups (*p* > 0.05). Our findings were consistent with earlier research. The novel measurement method will offer a new perspective on breastfeeding.

## 1. Introduction

The rooting, sucking, and swallowing reflexes of a healthy neonate become active shortly after birth. These reflexes, commonly referred to as sucking physiology, emerge during the prenatal period of development. A newborn infant possesses the innate ability to execute the sucking reflex without any difficulty. However, they must acquire the requisite abilities to effectively utilize these reflexes. The process of milk ejection from the breast is considered a learned ability that necessitates the proper attachment of the infant’s mouth to the breast and the comprehensive adaptation of anatomical structures, such as the tongue and lips, for feeding [1]. To achieve successful breastfeeding, the infant must possess the capability to effectively and securely ingest the milk bolus before it enters the digestive system. To execute this particular function, the infant must possess the capability to synchronize the processes of swallowing and respiration, all the while ensuring the maintenance of cardiovascular stability [2]. Symptoms indicating dysfunctions in these processes include breast and nipple pain, challenges with latching and sucking, crying during breastfeeding, refusal of the breast, extended and frequent feeding, inadequate weight gain, crying due to insufficient satiety, and difficulties with milk transfer [3]. Numerous challenges prompt a significant number of women to prematurely cease the practice of breastfeeding [4].

A comprehensive comprehension of the physiology of breastfeeding and an in-depth examination of the anatomical structures of the jaw, palate, hyoid bone, pharynx, and tongue involved in sucking, swallowing, and respiratory actions can enhance the efficacy of therapeutic interventions aimed at addressing breastfeeding difficulties experienced by newborns and mothers [5,6]. The execution of these functions is heavily reliant on the movements of the tongue [7,8]. The examination of literary works reveals the presence of two discernible perspectives regarding the movement of the tongue during the process of sucking. Based on the stripping action theory, the act of extracting milk from the breast is facilitated by peristaltic tongue movements and the application of sucking pressure through the infant’s mandible, compressing the breast [8,9,10,11,12]. According to the theory of intra-oral vacuum, the primary mechanism involved in the extraction of milk from the breast is the generation of negative pressure or a vacuum through the downward movement of the tongue [7,13]. Based on the findings of the study assessing milk outflow and sucking dynamics during breastfeeding, it was observed that the posterior tongue exhibits peristaltic movements, effectively guiding the milk bolus toward the esophagus. Furthermore, it should be noted that the anterior tongue tip, located beneath the nipple, applies pressure to the entire breast through periodic mandibular movements without exhibiting peristaltic motion [14]. There is a lack of consensus within the scientific literature regarding the specific role and function of the tongue during the lactation process.

The presence of anatomical variations in the structures involved in the act of sucking may potentially exert detrimental impacts on the process of breastfeeding. Ankyloglossia, also known as tongue tie, is a congenital anomaly that restricts the range of motion of the tongue. This particular condition exerts a significant impact on the suction capacity and may also modify the structure of the dental arches, consequently influencing occlusion [1,15,16]. The occurrence rate of ankyloglossia in newborns varies between 4% and 16%, with a greater frequency observed in males compared to females at a ratio of 2.5 to 1.2 [17]. Despite divergent opinions among healthcare professionals, the condition known as tongue tie has been widely acknowledged as a prevalent issue affecting breastfeeding, as evidenced by numerous studies. According to the findings of Messner et al. (2000), approximately 90% of pediatricians and 70% of otolaryngologists believe that ankyloglossia rarely causes feeding difficulties, while approximately 70% of lactation consultants reported that ankyloglossia often causes feeding difficulties [18]. Research findings indicate that ankyloglossia is linked to a substantial percentage of breastfeeding-related challenges in neonates, with prevalence estimates ranging from 25% to 60%. The aforementioned concerns encompass inadequate milk production, hindered growth and development, nipple injury, breast discomfort, engorgement, and maternal rejection [18,19,20]. The main contributing factor to these issues, as identified by Marmet et al. (2000) and Segal et al. (2007), is the neonate’s inability to maintain a secure latch on the breast, which can be attributed to ankyloglossia [20,21]. According to reports, the rate of breastfeeding cessation as a result of the enduring pain encountered by mothers during the initial three weeks of breastfeeding ranges from 10% to 26% [22,23]. The crucial factor for successful breastfeeding in healthy neonates, as determined through the use of ultrasonography, is appropriate tongue mobility, as indicated by the findings of Smith et al. (1985) [24]. In the case of neonates diagnosed with ankyloglossia, it has been observed that traditional positioning and suckling techniques do not yield satisfactory results in terms of restoring tongue mobility. Consequently, surgical intervention may become necessary [19]. In recent years, there has been an increasing prevalence of frenotomy procedures aimed at facilitating the continuity of breastfeeding in neonates. The existing literature does not provide a consensus on the effectiveness of frenotomy in lactation. There is still a lack of consensus regarding the appropriate course of action for addressing this abnormality, including the timing of treatment and the optimal surgical approach to be employed [25,26].

Therefore, implementing a comprehensive, accurate, and unbiased approach to assess the intraoral modifications occurring during the sucking reflex in newborns would effectively mitigate the need for unnecessary surgical interventions in early infancy. In their study, Geddes and Sakalidis (2016) presented a thorough elucidation of the ultrasound imaging technique employed for observing the tongue’s movements during the act of breastfeeding [27]. In their study, McClellan et al. (2010) utilized morphological metrics to illustrate the precise positioning of the nipple within the oral cavity and the tongue’s role during breastfeeding, as observed through B-mode ultrasonography images taken in the midsagittal plane [28].

The objective of this study was to conduct a comparative analysis between neonates diagnosed with clinically confirmed tongue ties and a control group of healthy individuals. The comparison was conducted in terms of tongue position and movement during sucking. Additionally, the study aimed to assess the effectiveness and applicability of ultrasound imaging techniques in this context. The main objectives of this study are to present a new and unbiased ultrasound diagnostic approach that has not yet been proven effective in the orofacial region and to establish innovative and unbiased diagnostic criteria to assist in the identification of neonates with tongue tie.

## 2. Materials and Methods

The research was carried out in compliance with the principles outlined in the Declaration of Helsinki and received approval from the Ethics Committee of Health Sciences at Ankara Yildirim Beyazit University in Ankara, Turkey (04/13 April 2023).

The sample size was calculated for the effect size (d, effect size = 0.85), type I error (α = 0.05), and 85% power values; the sample size was determined to be 30 for the two independent groups.

The current study entailed a retrospective examination of ultrasonography images acquired using B-mode (brightness mode) and M-mode (motion mode) techniques. These images were obtained from a cohort of sixty neonates between May 2022 and December 2022. The researchers conducted a review of the file records of the neonates included in this study, focusing on two key pieces of information: the severity of tongue tie, as assessed using the Bristol Tongue Assessment Tool, and whether a frenotomy procedure had been carried out. Based on the scoring system of the Bristol Tongue Assessment Tool, the participants were categorized into two distinct groups: a group of 30 individuals with ankyloglossia, characterized by a tongue tie score of 5 or less, and a group of 30 healthy individuals, characterized by a tongue tie score exceeding 5. During the course of breastfeeding, ultrasonography images were obtained from full-term neonates aged 5–15 days. All USG evaluations were conducted by a single dentomaxillofacial radiologist observer with 14 years of experience (A.A). The radiologist employed a GE Medical System Verasana Active (Wuxi, Jiangsu, China) mobile ultrasonography device equipped with a 6–10 MHz 8C-RS microconvex probe. This instrument was used to capture 2D real-time, B-mode, and M-mode images of the oral cavity of the neonates. The imaging was performed using a submental approach in the midsagittal plane. The examinations were conducted using Parker Ultrasonic Gel (Fairfield, NJ, USA). The data collection commenced when the infant began to latch onto the breast and concluded when the feeding came to an end. The recording captured by the scan was specifically intended for subsequent academic investigation. The study excluded recordings that exhibited artifacts resulting from movements of the mother–newborn pair or the operator, as well as recordings in which the presence of nutritive sucking could not be observed. All measurements were conducted in recorded ultrasonography images. Before starting the measurement in the study, the observer was calibrated to recognize as well as to identify the neonate maxillofacial and oral anatomy; for such purpose, 10 different neonate ultrasonography images other than the study were used. The observer was blinded to any patient data. 

**Figure 1:**
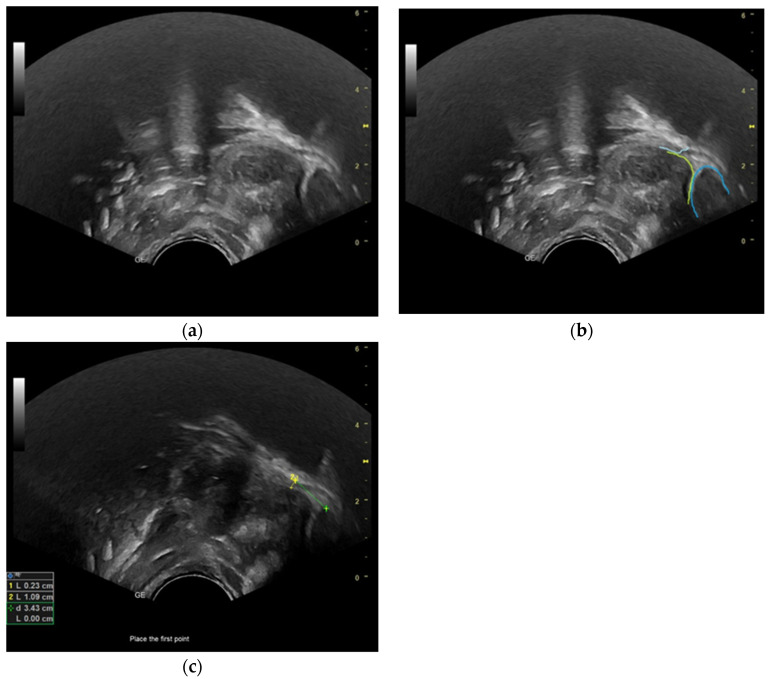
Ultrasonographic image of breastfeeding: (**a**) nipple view, tongue up; (**b**) nipple: dark blue line, hard–soft palate junction (HSPJ): light blue line, tongue: yellow line; (**c**) depth of intraoral space, cm: yellow line 1; nipple–HSPJ distance, cm: green line 2.

**Figure 2:**
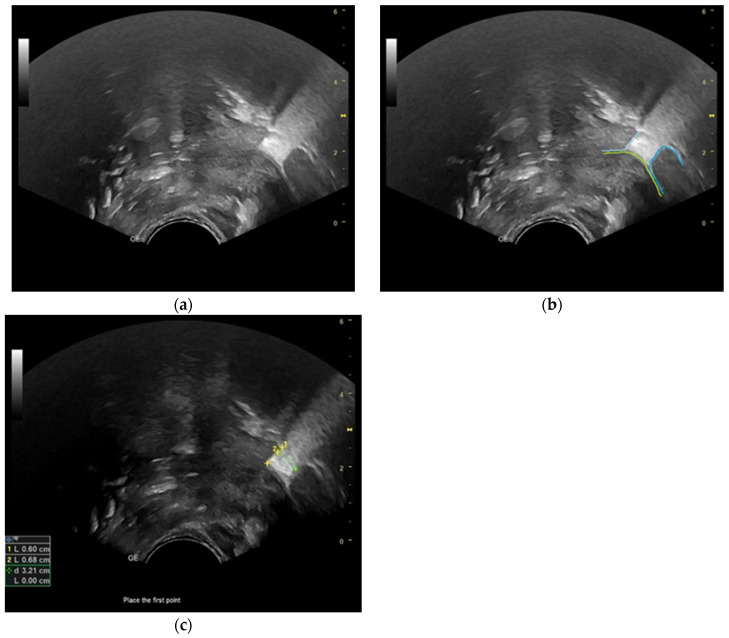
Ultrasonographic image of breastfeeding: (**a**) nipple view, tongue down; (**b**) nipple: dark blue line, HSPJ: light blue line, tongue: yellow line; (**c**) depth of intraoral space, cm: yellow line 1; nipple–HSPJ distance, cm: green line 2.

During the act of breastfeeding, the nipple is positioned in such a way that it rests on the anterior region of the tongue, while the tip of the anterior tongue is elevated above the inferior alveolar ridge. The visibility of the tip of the tongue on ultrasonography is hindered by mandibular superposition. The process of sucking begins with the tongue-up posture (Figure 1a–c), wherein the middle portion of the tongue is near the roof of the mouth. This occurs when a newborn is properly attached to the breast and the tongue occupies the entire oral cavity. The sucking cycle is characterized by a downward motion of the tongue towards its lowest point, known as the tongue-down position (Figure 2a–c). In this position, the middle portion of the tongue descends to the floor of the mouth. The cycle concludes when the tongue reverts to its original position adjacent to the palate. The downward movement of the tongue results in the flow of breast milk into the interstitial space created between the surface of the tongue and the palate. In the ultrasonography image, milk boluses are observed as areas of increased echogenicity within the hypoechoic region [28]. Upon the tongue’s elevation towards the palate, the milk bolus is introduced into the pharynx. The ultrasound image depicts the process of sucking, where milk is transferred into the oral cavity, referred to as nutritive sucking (Figure 2a–c). Conversely, the act of sucking without milk outflow is termed non-nutritive sucking [29].

**Figure 3:**
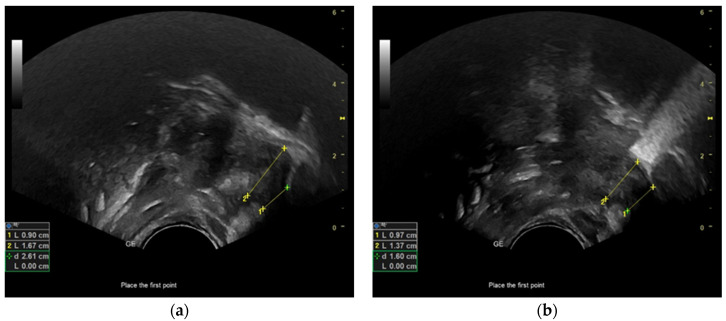
Ultrasonographic image of the tongue during breastfeeding: (**a**) tongue up; anterior tongue height, mm: yellow line 1; posterior tongue height, mm: yellow line 2; (**b**) tongue down; anterior tongue height, cm: yellow line 1; posterior tongue height, cm: yellow line 2.

Frame-by-frame analysis of B-mode ultrasonography recordings was conducted to examine three consecutive sucking cycles during nutritive sucking in both groups. The determination of the nipple’s position in the mouth during each cycle involved measuring the distance between the HSPJ and the nipple as well as the depth of the intra-oral space created during the sucking function, as outlined by McClellan et al. (2010) (Figure 1c and Figure 2c) [28]. In ultrasonography images, the hard palate is visualized as a line with high echogenicity, while the soft palate is observed as a structure of intermediate gray shades with a distinct echogenic upper boundary (see Figure 1b and Figure 2b). The observation of the soft palate’s motion during the sucking process facilitated the identification and validation of the HSPJ through the analysis of real-time ultrasonography video footage. To determine the alteration in size of the tongue during the act of sucking, measurements of the anterior and middle sections of the tongue were taken in both the tongue-up and tongue-down orientations. The researchers obtained measurements from the dorsal surface of the tongue to the inferior border of the genioglossus muscle, as depicted in Figure 3a,b.

**Figure 4:**
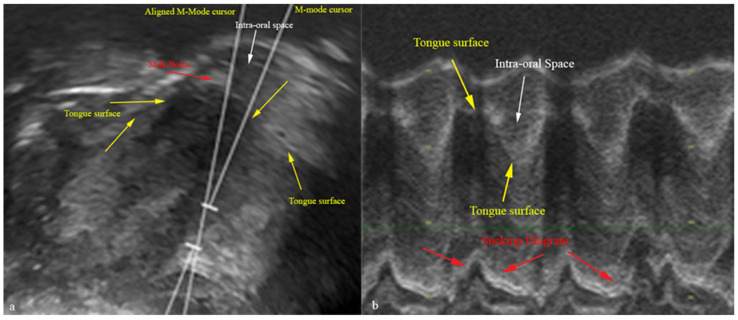
Ultrasound sonographic image of the tongue movement during sucking cycle in B-mode and M-mode. (**a**) The yellow arrow shows the tongue surface, the red arrow milk bolus, the white arrow intra-oral space, and white lines show M-mode cursor lines; (**b**) the yellow arrow shows the tongue surface, the red arrow shows the sucking cycle diagram, and white arrow intra-oral space.

**Figure 5:**
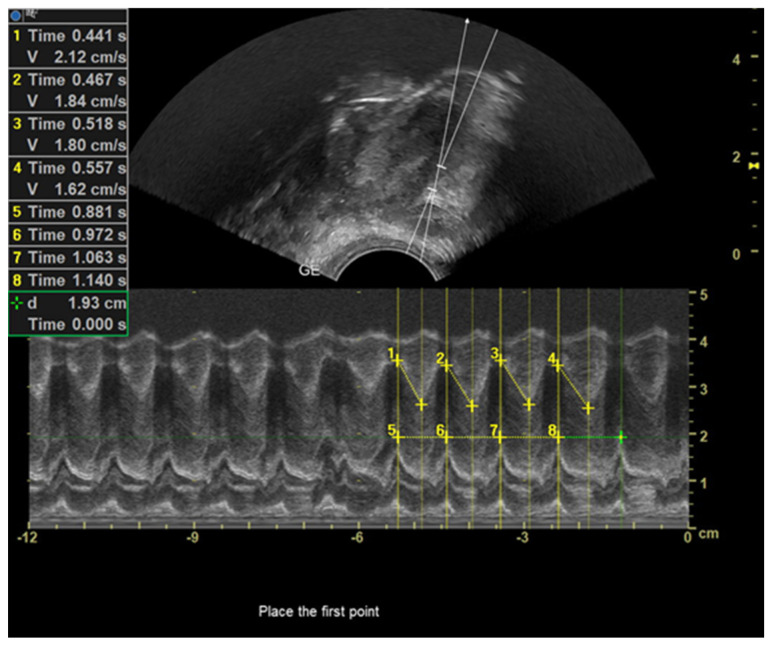
M-mode ultrasonography of tongue movement during breastfeeding. Slope measurement: yellow line 1, 2, 3 and 4; sucking cycle duration: yellow line 5, 6, 7 and 8.

In this study, we employed the measurement technique previously employed by Schleifer et al. (2021) and Boussuges et al. (2020) to evaluate tongue movements and the duration of a sucking cycle during breastfeeding [30,31]. The M-mode cursor was placed at a perpendicular angle to the mid-tongue in ultrasonography images acquired from both groups of neonates utilizing anatomical M-mode. The utilization of anatomical M-mode imaging resulted in improved visualization of the echogenic representation of tongue motility (Figure 4a,b). In M-mode tongue movement images obtained during nutritive feeding, the tongue is hypoechoic. The milk bolus is viewed as hyperechoic on the hypoechoic image of the tongue. In this diagram, the initial caliper was designated at the apex corresponding to the placement of the tongue, while the subsequent caliper was designated at the nadir of the incline formed by the tongue’s downward motion. Ultrasonographic measurements were conducted to assess tongue excursion from the palate to the floor of the mouth (in centimeters), excursion time (in seconds), and tongue velocity during movement (in centimeters per second). Furthermore, the initial caliper was positioned at the apex of the hypoechoic diagram representing tongue movement, specifically when the tongue was situated above. Similarly, the second caliper was placed at the subsequent peak of the hypoechoic diagram, also indicating tongue positioning above. Subsequently, the duration (in seconds) of a single sucking cycle was determined. The mean values of the data obtained in M-mode were determined by conducting repetitive measurements over four sucking cycles (Figure 5). Using this novel ultrasonographic measurement technique, the researchers determined the mean duration of the nutritive sucking cycle and the average velocity of tongue movement. The analysis of frenotomy status among neonates diagnosed with tongue tie in our study group was conducted concerning the remaining parameters.

All measurements were taken twice by the same observer, and the mean values of all measurements were included in the statistical analysis. The observer also performed the study twice with an interval of 2 weeks to detect intra-observer variability. Intra-observer reliability was conducted. To assess intra-observer reliability, the Wilcoxon matched pairs signed rank test was used for repeat measurements.

The data for this study were analyzed utilizing IBM SPSS Statistics V22.0 (New York, NY, USA). The Shapiro–Wilk test was utilized to assess normality. The variables nipple-HSPJ distance intraoral space depth, anterior and mid-tongue height, tongue excursion from the palate to the floor of the mouth (cm), excursion time (sec), tongue velocity (cm/sec), and one sucking cycle duration (sec) obtained in the tongue tie and control groups exhibited a non-normal distribution. Therefore, the Mann–Whitney U test was employed to assess the association between the two groups. The frenotomy status data were subjected to a *t*-test to compare them with other variables as they exhibited a normal distribution. The values are presented in terms of the mean and standard deviation (SD). The chosen level of significance for this study was 0.05. A *p*-value less than 0.05 is indicative of a statistically significant difference or correlation, while a *p*-value greater than 0.05 suggests the absence of a statistically significant difference or correlation.

## 3. Results

Repeated measurements indicated no significant intra-observer difference for the observer (*p* > 0.05). Overall intra-observer consistency was rated among the measurements between 92 and 95%. All measurements were found to be highly reproducible for the observer and no significant difference was obtained from two measurements of the observer (*p* > 0.05). Thus, the mean values of these measurements from the three points were considered to be the final data.

The present study analyzed ultrasonography images obtained from a sample of 30 neonates who were diagnosed with ankyloglossia. This sample consisted of 13 female neonates and 17 male neonates. The mean age of the neonates in this study was determined to be 12 days. By contrast, the mean age of a sample of 30 neonates who were in good health was found to be 11 days.

The findings from our examination of ultrasonography images indicate that, during the process of newborns attaching to the breast, the tongue assumes a resting position on the palate in conjunction with the nipple. The nutrient extraction process began in a state of quiescence. The observation of anechoic milk ducts in the nipple and the subsequent movement of the nipple towards the HSPJ can be attributed to the downward displacement of the middle tongue. At the point of closest proximity between the nipple and the HSPJ, the tongue reached its lowest position while the intraoral space was filled with milk. The movement of the milk bolus towards the pharynx was noted when the middle tongue returned to the palate, causing the cessation of milk flow due to the pressure exerted by the tongue on the nipple. Upon adjusting the position of the tongue against the palate, it was noted that the nipple exhibited a displacement away from the HSPJ in both experimental groups. The data collected in this study were employed to ascertain the location of the nipple within the oral cavity as well as the dimensional fluctuations in the vertical movements of the tongue during the sucking process for both experimental groups.

In the tongue-up position during the sucking cycle, it was observed that, in the ankyloglossia group, the nipple exhibited a greater distance from the HSPJ compared to the control group (*p* < 0.05) (Table 1). There was a statistically significant difference in the anterior tongue height in the tongue-up position between the control group and the ankyloglossia group (*p* < 0.05) (Table 1). In contrast to neonates diagnosed with ankyloglossia, neonates in the control group demonstrated greater ability to elevate the anterior tongue in proximity to the palate during the act of sucking. While there was no statistically significant disparity observed between the two groups regarding anterior tongue height in the tongue-down position, it was observed that healthy neonates exhibited greater anterior tongue height compared to those diagnosed with ankyloglossia (*p* > 0.05). The neonates in the control group exhibited greater proficiency in the act of latching onto and maintaining the breast within their oral cavity compared to the neonates in the ankyloglossia group, as indicated by the data presented in Table 1. Both groups exhibited similar measurements for intraoral space depth and mid-tongue height, as shown in Table 1.

This study aimed to assess the tongue movements observed during the sucking cycle. There was no statistically significant difference observed between neonates diagnosed with ankyloglossia and those in the control group in terms of the duration of tongue movement from the palate to the floor of the mouth, the pace of the tongue during this movement, or the duration of a sucking cycle (*p* > 0.05; Table 2).

A decision was made to conduct frenotomies on 16 out of 30 neonates diagnosed with ankyloglossia based on the presence of ultrasonography records and the persistence of breastfeeding difficulties. The average score for the lingual frenulum in neonates who received frenotomy was 3.75 (±1.06), while the average score for neonates who did not receive frenotomy was 4.29 (±1.14) (*p* > 0.05) (Table 3). There was no statistically significant difference observed in the scores between the two groups. Furthermore, there was no statistically significant distinction observed between neonates who received intervention and those who did not regarding the extent of nipple approach to the HSPJ, depth of intraoral space, or heights of the tongue (*p* > 0.05) (Table 4). When assessing the variables related to the duration of tongue displacement from the palate to the floor of the mouth, the velocity of the tongue during this motion, and the duration of a complete sucking cycle, no statistically significant distinction was observed between two groups of neonates (*p* > 0.05) (Table 5).

## 4. Discussion

This study introduces a novel and objective measurement technique for assessing tongue movements in neonates during breastfeeding.

Furthermore, we evaluated the differences between neonates with ankyloglossia and healthy neonates regarding the positioning of the nipple within the oral cavity and the positioning and movements of the tongue during the sucking process. Additionally, we compared these parameters between individuals in the ankyloglossia group who underwent frenotomy and those who did not.

According to a study conducted by Li et al. (2008), the primary factor leading to the cessation of early breastfeeding is the challenge faced by neonates in terms of sucking and latching onto the breast [4]. However, at present, there is a lack of a clinically feasible and objective measurement method that elucidates the intricacies of sucking action, specifically the movements of the tongue.

The measurement techniques in the literature that utilize ultrasonography to diagnose based on the extent of muscle movement have garnered considerable interest [30,31]. The applicability of these techniques to the orofacial region has not yet been demonstrated. The studies conducted by Schleifer et al. (2021) and Boussuges et al. (2020) employed the M-mode imaging technique to evaluate the functionality of the diaphragm during both expiration and inspiration. To assess the rate of displacement of the diaphragm during expiration or inspiration, the researchers placed the initial caliper at the lower end of the diaphragmatic echoic slope generated by diaphragmatic motion and the second caliper at the highest point of the slope. As a result, the researchers computed the diaphragmatic excursion in centimeters (cm) during both inspiration and expiration. They also determined the duration of the excursion in seconds (s) and the mean velocity of the excursion in centimeters per second (cm/s). Furthermore, the authors showcased the temporal extent of a single respiratory cycle, encompassing both the inhalation and exhalation phases [30,31]. 

In our study, adjustments were made to the measurements on M-mode ultrasonography recordings to account for the movements of the tongue during the sucking cycle. The introduction of this novel measurement technique is expected to offer an objective and replicable means of assessing the physiological aspects of sucking. This technique enables the interpretation of both the duration and velocity of tongue displacement during the sucking function. This study utilized a novel measurement technique to compare the parameters of tongue movement between neonates diagnosed with ankyloglossia and neonates who were deemed healthy. Based on the findings, there was no discernible disparity observed in the rate and duration of lingual movements among neonates diagnosed with ankyloglossia in comparison to those belonging to the control group. The results of this study indicate that ankyloglossia does not have a detrimental impact on the vertical movement of the tongue or the flow of milk in neonates. In their research on the effectiveness of ultrasonography studies in examining breastfeeding and sucking dynamics, Douglas et al. (2018) observed that the tongue and mandible exhibit synchronized movement without any independent motion. Furthermore, they found that the lingual frenulum does not exert any influence on the tongue’s movement during sucking [3]. The outcome obtained confirms the conclusions drawn from our research. In a study conducted by Geddes et al. (2008), an examination was carried out on tongue movements during breastfeeding. The researchers discovered that the downward motion of the tongue creates a suction effect on the nipple, resulting in the release of milk from the breast [32]. Elad et al. (2014) conducted a biomechanical investigation to examine the mechanics of sucking. Their study revealed the coordinated movement of the anterior tongue with the mandibular motion, specifically positioning itself beneath the nipple. Additionally, the middle and posterior portions of the tongue were found to generate a vacuum effect, facilitating the outflow of milk [14]. The results suggest that the anterior tongue’s role in the sucking process may be more pronounced and effective in achieving the necessary oral isolation needed to create a vacuum, thereby aiding in the attachment to the breast and the ejection of milk. In their study, Geddes et al. (2021) conducted a comprehensive examination of breastfeeding and observed that the application of frenotomy to the anterior tongue tie resulted in an increase in milk output and a decrease in nipple pain. Conversely, the application of frenotomy to the posterior tongue tie did not have any impact on milk production but did lead to a reduction in nipple pain [33]. When assessing the severity of ankyloglossia and the necessity of frenotomy, it is hypothesized that conducting further investigations to examine the impact of tongue extension towards the anterior crest of the mandible on breastfeeding latch and sucking functionality would offer a novel insight into tongue function.

Tongue movement was assessed using M-mode in the context of these innovative ultrasonography measurements. Previous studies have reported that there may be a discrepancy between the M-mode cursor and the true axis of the structure being imaged. This discrepancy has raised concerns regarding the potential for inaccurate measurements [30]. Ensuring axis alignment during real-time ultrasonography on breastfeeding neonates poses a significant challenge, primarily attributable to the inherent difficulty in controlling the movements of both the mother and the infant. The angle-independent M-mode, also known as anatomical or post-processing M-mode, allows for unrestricted rotation and movement of the M-mode cursor to capture M-mode images from any desired angle [30]. In the present study, the determination of the examination line was conducted utilizing anatomic M-mode, whereby the cursor was positioned along the vertical axis of the tongue in its elevated position. This enhancement resulted in improved visual clarity of M-mode measurement images.

In their study, Jacobs et al. [34] conducted an investigation using diverse ultrasound transducers to observe the intraoral anatomy of infants during breastfeeding. Their findings led them to determine that a frequency range of 8–10 MHz would be adequate for infants under 12 weeks of age in the context of evaluating contemporary technological devices. In the aforementioned study, the researchers also underscored the appropriateness of convex long-handled transducers (specifically endocavity transducers) for assessing intraoral tissues while the subject is engaged in sucking activities. In our investigation, we opted for a 6–10 MHz 8C-RS microconvex probe that possesses imaging capabilities comparable to endocavitary probes. The image recordings were conducted at a frequency of 8 MHz. The study revealed that the microconvex probe, characterized by its expansive visual scope and elevated frequency spectrum, effectively captures images of intraoral tissues during breastfeeding. Moreover, its compact, convex surface and slender grip area make it particularly suitable for imaging in confined spaces.

According to the findings of Douglas et al. (2018) and Elad et al. (2014), it has been observed that, in healthy neonates, during the sucking process, the anterior tongue tip is situated below the nipple and on the inferior alveolar ridge [3,14]. The analysis of B-mode ultrasonography images revealed that healthy neonates, when in the tongue-up position, exhibited a closer proximity of the nipple to the HSPJ and demonstrated a more pronounced anterior expansion of the tongue compared to neonates with ankyloglossia. There was no statistically significant difference observed in the measured parameters between neonates with ankyloglossia and those without this condition. Based on the findings of our study, it was observed that neonates with ankyloglossia possess the ability to bring the nipple closer to the HSPJ at a comparable rate to that of healthy neonates. This phenomenon occurs due to the creation of a vacuum within the oral cavity when breastfeeding in the tongue-down position. When the tongue is elevated, the loss of the nipple’s position in the mouth occurs as a result of a decrease in oral vacuum. However, in neonates with ankyloglossia, the nipple moves further away from the HSPJ compared to healthy neonates. It is hypothesized that the presence of ankyloglossia in neonates hinders their ability to extend their tongue toward the inferior alveolar ridge. Thus, it is postulated that they encounter challenges in successfully latching onto the breast and maintaining proper nipple placement within the oral cavity, consequently leading to frequent disruption of the initial sucking required for proper attachment. Based on the ultrasonography measurements and the observations conducted on a substantial sample size within our study, it has been determined that the anterior tongue exhibits an active role in the retention of the nipple within the oral cavity. There is a belief that conducting additional research on the role of the anterior tongue can provide insights into the challenges faced by neonates with ankyloglossia in latching onto the breast as well as the early breast pain experienced by mothers.

Previous studies have presented divergent viewpoints regarding the requisite nature of frenotomy and its impact on sucking and latching difficulties in neonates diagnosed with ankyloglossia [25,26]. A subset of the neonates whose images were utilized in our study were found to necessitate frenectomies as a result of enduring difficulties with breastfeeding. The pre-procedural ultrasonography measurements of neonates who underwent frenotomy and those who did not undergo frenotomy were found to be statistically similar. This suggests that there were no significant disparities in tongue movements that would influence the decision to pursue surgical intervention. While the diagnosis of ankyloglossia typically indicates the need for a frenotomy procedure, it is important to consider the impact of neonatal development and the family’s decision making process on the effectiveness of this intervention. The level of objectivity regarding the decision remains uncertain.

The present study is subject to several limitations. The inability to conduct follow-up assessments on neonates with ankyloglossia, both with and without frenotomy, was a result of the study’s retrospective design. Hence, the impact of surgical intervention on breastfeeding outcomes, as assessed through the use of ultrasound imaging, could not be ascertained. The absence of both short- and long-term follow-up studies creates uncertainty regarding the persistence of breastfeeding difficulties associated with ankyloglossia. The examination of subsequent records within this study will enable us to effectively illustrate the effectiveness of the novel ultrasonography measurement technique employed in the oral region.

The implementation of further research endeavors aimed at assessing the effectiveness of frenotomy will furnish healthcare professionals with essential benchmarks for making informed decisions regarding diagnosis and treatment. Additionally, these studies will yield practical diagnostic instruments that can be readily employed to evaluate nipple pain in mothers and breastfeeding difficulties, such as impaired latching and sucking, in neonates diagnosed with ankyloglossia. Consequently, neonates will be safeguarded from undergoing unnecessary surgical procedures at an early stage.

## 5. Conclusions

This study presents a novel approach in the field of ultrasonography measurement techniques, which offers a valuable and applicable means of objectively evaluating tongue function in the context of breastfeeding. The measurement methodology utilized produced findings that aligned with the outcomes of prior research. There is no significant difference in tongue movements in the middle tongue region between neonates diagnosed with ankyloglossia and neonates who are considered healthy. This methodology enables the assessment of different areas of the tongue and other movable organs implicated in the sucking process during breastfeeding.

## Figures and Tables

**Table 1:** Nipple and tongue measurements. Ankyloglossia and control group comparisons.

			Nipple–HSPJ Distance, cm	Depth of Intraoral Space, cm	Anterior Tongue Height, cm	Mid-Tongue Height, cm
Tongue up	Ankyloglossia	n	30 *	30	30 *	30
		mean	0.81 *	0.33	1.08 *	1.66
		SD	0.32 *	0.13	0.29 *	0.26
	Control	n	30 *	30	30 *	30
		mean	0.64 *	0.28	1.26 *	1.76
		SD	0.18 *	0.08	0.23 *	0.18
Tongue down	Ankyloglossia	n	30	30	30	30
		mean	0.51	0.74	1.06	1.33
		SD	0.25	0.29	0.18	0.21
	Control	n	30	30	30	30
		mean	0.43	0.43	1.17	1.36
		SD	0.23	0.26	0.2	0.22

* Statistical analysis performed using Mann–Whitney U test (*p* < 0.05). n: number of participants; SD: standard deviation.

**Table 2:** Tongue movement variables. Ankyloglossia and control group comparisons.

		The Velocity of Transition from the Palate to the Mouth’s Floor, cm/s	The Duration of the Transition from the Palate to the Mouth’s Floor, s	Sucking Cycle Duration, s
Ankyloglossia	n	30	30	30
	mean	2.07	0.44	0.77
	SD	0.65	0.15	0.19
Control	n	30	30	30
	mean	2.03	0.44	0.81
	SD	0.44	0.13	0.19

Statistical analysis performed using Mann–Whitney U test (*p* < 0.05). n: number of participants; SD: standard deviation.

**Table 3:** Lingual frenulum score and frenotomy decision.

		Lingual Frenulum Score
Frenotomy (−)	n	14
	mean	4.29
	SD	1.14
Frenotomy (+)	n	16
	mean	3.75
	SD	1.06

Statistical analysis performed using Mann–Whitney U test (*p* < 0.05). n: number of participants; SD: standard deviation.

**Table 4:** Nipple–tongue measurements and frenotomy decision groups comparisons.

			Nipple–HSPJ Junction Distance, cm	Depth of Intraoral Space, cm	Anterior Tongue Height, cm	Mid-Tongue Height, cm
Tongue up	Frenotomy (−)	n	14	14	14	14
		mean	0.81	0.36	1.18	1.64
		SD	0.31	0.15	0.23	0.21
	Frenotomy (+)	n	16	16	16	16
		mean	0.82	0.31	1	1.71
		SD	0.34	0.10	0.32	0.31
Tongue down	Frenotomy (−)	n	14	14	14	14
		mean	0.43	0.82	1.08	1.28
		SD	0.16	0.31	0.17	0.20
	Frenotomy (+)	n	16	16	16	16
		mean	0.57	0.68	1.05	1.37
		SD	0.3	0.28	0.19	0.21

Statistical analysis performed using Mann–Whitney U test (*p* < 0.05). n: number of participants; SD: standard deviation.

**Table 5:** Tongue movement variables and frenotomy decision groups comparisons.

		The Velocity of Transition from the Palate to the Mouth’s Floor, cm/s	The Duration of the Transition from the Palate to the Mouth’s Floor, s	Sucking Cycle Duration, s
Frenotomy (−)	n	14	14	14
	mean	2.16	0.47	0.82
	SD	0.68	0.18	0.18
Frenotomy (+)	n	16	16	16
	mean	2	0.41	0.73
	SD	0.63	0.12	0.19

Statistical analysis performed using Mann–Whitney U test (*p* < 0.05). n: number of participants; SD: standard deviation.

## Data Availability

The datasets generated and analyzed during the study are available from the corresponding author upon reasonable request.
