# Correction: Alan et al. Evaluation of the Breastfeeding Dynamics of Neonates with Ankyloglossia via a Novel Ultrasonographic Technique. *Diagnostics* 2023, *13*, 3435

**DOI:** 10.3390/diagnostics14060608

**Published:** 2024-03-13

**Authors:** Arzu Alan, Ayse Isil Orhan, Kaan Orhan

**Affiliations:** 1 Ankara 75th Year Oral and Dental Health Hospital, Ministry of Health, Ankara 06230, Türkiye; arzudogruyol@yahoo.com; 2 Department of Pediatric Dentistry, Faculty of Dentistry, Ankara Yildirim Beyazit University, Ankara 06220, Türkiye; isilcihan@yahoo.com; 3 Department of Dentomaxillofacial Radiology, Faculty of Dentistry, Ankara University, Ankara 06560, Türkiye

There were a few errors in the original publication [1]. 

**1.** A correction has been made to the 2nd sentence of paragraph 8, in the Materials and Methods section, as follows: The variables nipple-HSPJ distance intraoral space depth, anterior and mid-tongue height, tongue excursion from the palate to the floor of the mouth (cm), excursion time (sec), tongue velocity (cm/sec), and one sucking cycle duration (sec) obtained in the tongue tie and control groups exhibited a non-normal distribution.

**2.** The corrected version of the 2nd sentence of paragraph 6, in the Results section is as follows: The average score for the lingual frenulum in neonates who received frenotomy was 3.75 (±1.06), while the average score for neonates who did not receive frenotomy was 4.29 (±1.14) (*p* > 0.05) (Table 3).

The authors state that the scientific conclusions are unaffected. This correction was approved by the Academic Editor. The original publication has also been updated.
